# Genetic analysis of three wild Eurasian eagle-owl subspecies, *B. b. kiautschensis*, *B. b. ussuriensis,* and *B. b. tibetanus,* in Chinese populations

**DOI:** 10.1080/23802359.2020.1839363

**Published:** 2020-11-23

**Authors:** Meng Meng, Jianzhang Ma, Muhammad Younis Laghari, Jianwei Ji

**Affiliations:** a College of Wildlife and Protected Area, Northeast Forestry University, Harbin, China; b China Wildlife Conservation Association, Beijing, China; c Department of Freshwater Biology and Fisheries, University of Sindh, Jamshoro, Pakistan; d Beijing Wildlife Rescue and Rehabilitation Central, Beijing, China

**Keywords:** *Bubo bubo*, mitochondrial genome, molecular phylogeny

## Abstract

The Eurasian eagle-owl (*Bubo bubo*) is distributed throughout Asia and Europe and contains approximately twelve subspecies. Three subspecies, *B. b. kiautschensis, B. b. ussuriensi*s, and *B. b. tibetanus,* are separately distributed in the refugia and plateau habitats of China. However, the genetics of these subspecies and populations have not been studied. Genetic differences were investigated among 32 individuals from six populations of these three *B. bubo* subspecies based on the mitochondrial genome. Low genetic diversity but high haplotype diversity was observed in these subspecies. The phylogenetic relationship of three *B. bubo* subspecies distributed in China was proven to be coordinated with geographic and environmental gradients. This study provides the first detailed insights into the mitochondrial genetic diversity of three Eurasian eagle-owl subspecies distributed in China and demonstrates the utility of the mitochondrial genome in intraspecific genetic population analyses of these eagle-owls.

The Eurasian eagle-owl (*Bubo bubo*) is a large owl that occurs in a wide range of habitats in Asia and Europe (Kleven et al. 2013); approximately twelve subspecies are recognized. Three subspecies, *B. b. kiautschensis, B. b. ussuriensi*s, and *B. b. tibetanus,* are separately distributed in the complex geographic habitat of China. *B. b. kiautschensis is* distributed in South China (SC), which is one of the country’s species richness hotspots. It has been proposed that the topographical complexity of this region would provide stable habitats during ice ages (Hewitt 2000). SC is not only a glacial refugium but also an area connected to the North China (NC) regions with a distribution of *B. b. ussuriensis* and to the Qinghai-Yunan Plateau of China (QYC) regions with a distribution of *B. b. tibetanus* (Zheng 2005, 2011). Thus, the high geographical complexity of these regions could complicate the genetic diversity and structure of the three subspecies (Lu and Ma 2010). However, the genetics of these subspecies and populations have not been studied in the past.

To provide the first look into the genetic variation and demographic processes of *B.b. kiautschensis, B. b. ussuriensi*s, and *B. b. tibetanus* dispersed throughout China, we employed complete mitochondrial genome analysis to evaluate the genetic diversity and genetic structure of these three subspecies. A total of 32 feather samples of adult *B. bubo,* including individuals of all three subspecies, were collected from six Chinese locations: Hunan (HN) and Hubei (HB) populations of *B. b. kiautschensis*; Qinghai (QH) and Yunnan (YN) populations of *B. b. tibetanus*; and Beijing (BJ) and Heilongjiang (HLJ) populations of *B. b. ussuriensis* (Figure 1(A), Table 1). The feather specimens (from BWRR-Bu-2018-001 to BWRR-Bu-2018-032) are stored in the zoology specimen collection of the Beijing Wildlife Rescue and Rehabilitation Central Lab, Beijing, China.

**Figure 1.**
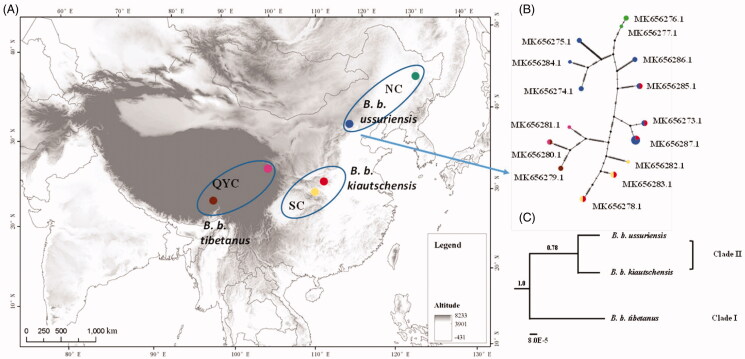
Map and phylogenetic analysis of 32 *B. bubo* mitogenomes. (A) Map indicating the collection sites for 32 *B. bubo* samples. (1) *B. b. ussuriensis*, NEC lineage, Heilongjiang (*n* = 3, green), Beijing (*n* = 14, blue); (2) *B. b. kiautschensis*, SC lineage, Hunan (*n* = 6, yellow), Hubei (*n* = 2, red); (3) *B. b. tibetanus*, QYC lineage, Qinghai (*n* = 4, brown), Yunnan (*n* = 3, pink). (B) Median-joining network obtained from 32 *B. bubo* mitogenomes. Sizes of circles are proportional to the frequencies of haplotypes. Colors define the geographical origins of the samples based on subspecies distribution. (C) Phylogenetic tree derived from Bayes based on three *B. bubo* subspecies complete mitogenomes. Posterior probabilities are presented above branches.

**Table 1.** Information, genetic statistics, and neutrality tests for samples.

*B. bubo* subspecies	Sample site	Geographical coordinates	Sample size	Collecting date	V	H	HD	*π*	Tajima *D*	Fu's*F*s
*B. b. ussuriensis*	Beijing	40°33′N−116°49′E	14	2012–2018	116	9	0.91 ± 0.04	0.0015 ± 0.00001	−0.94_*p* > 0.1	4.47_*p* > 0.01
	Heilongjiang	45°21′N−127°28′E	3	2014–2018						
*B. b. kiautschensis*	Hubei	30°31′N−114°26′E	2	2016–2018	114	6	0.89 ± 0.07	0.0018 ± 0.00002	−0.74_*p* > 0.1	6.25_*p* > 0.01
	Hunan	28°10′N−112°56′E	6	2016–2018						
*B. b. tibetanus*	Yunnan	28°16′N–99°05′E	3	2015–2018	46	3	0.70 ± 0.04	0.0009 ± 0.00002	−0.03_*p* > 0.1	5.87_*p* > 0.01
	Qinghai	36°44′N–100°24′E	4	2015–2018						

V: number of polymorphic sites; H: number of haplotypes; HD: haplotype diversity; *π*: nucleotide diversity.

We extracted genomic DNA using QIAGEN DNeasy kits (QIAGEN, Inc.) from a single feather calamus and amplified the mitochondrial DNA genome using a set of seven primer pairs (Kang et al. 2018) (Table S1). Haplotype diversity (ĥ) and nucleotide diversity (π) were calculated with DnaSP v.5.0 (Librado and Rozas 2009). The genetic differentiation between populations and analysis of molecular variance (AMOVA) was calculated by Arlequin ver.2.0 (Excoffier and Lischer 2010) with 10,000 permutations. To reveal the phylogenetic relationship of the three subspecies, the median-joining haplotype network was constructed using Popart 1.7 software (Bandelt et al. 1999). In addition, phylogenetic trees for subspecies were created using the complete mitogenome. All individuals were grouped by subspecies, and each subspecies of mitogenomes was used as a separate operational population unit (OPU). The phylogenetic tree was executed using the Bayesian algorithm in BEAST with the HKY substitution model and the gamma and invariant-site heterogeneity models using OPU (version 1.8.0) (Drummond and Rambaut 2007).

The mitogenome sizes of these individuals of *B. b. kiautschensis and B. b. ussuriensis* were determined to be 18,951, 18,952, and 18,954 bp, and *B. b. tibetanus* was 18,951 and 18,954 bp. Fifteen haplotypes were found among these three subspecies (GenBank Accession: MK656273-MK656287). The subspecies *B. b. kiautschensis* contained an ancestral haplotype (Hap._9 of *B. b. kiautschensis,* MK656827) with a cumulative frequency of 28%.

The mitogenome of *B. bubo* contained the typical 13 PCGs, 22 tRNA genes, two rRNA genes, and two control regions (CRs). Duplicate CR copies were observed in all of the tested individuals, and there was a moderate sequence similarity (75–94%) between the two copies. Interestingly, we did observe 1- and 3-bp deletions/inserts in three loci in CR1 and 1-, 2- and 4-bp deletions/insertions in three loci in CR2, all of which were not located in CR-specific conserved blocks. In general, the structure of the mitochondrial genome was found to be conserved among the three subspecies by sequencing the whole mitogenomes of 32 individuals.

Protein-coding genes of the mitogenome separately produced 1–22 polymorphic sites in all of the tested individuals, among which 20 sites were synonymous and 30 sites were non-synonymous. The ATP8 gene and most of the tRNA-coding genes were not polymorphic within the studied samples, and only tRNA-Phe, tRNA-Val, and tRNA-Glu were variable in these individuals (Table S2).

High haplotype diversity and low nucleotide diversity were observed in these subspecies of *B. bubo*. Genetic analysis showed that the three lineages had a high level of haplotype diversity (0.70–0.91) and a low level of nucleotide diversity (0.0009–0.0018) estimated based on the mitogenome sequences (Table 1). *Bubo bubo kiautschensis* had the highest average nucleotide diversity.

AMOVA was performed to further examine the genetic structures of *B. b. ussuriensis* populations based on the *F_ST_* values. AMOVA revealed that the G1 method achieved the best result, showing the highest value of the source of variance among groups (21.81%) and the most significant genetic differences among groups (*F_CT_* = 0.20) (Table S3). The results illustrated genetic differences between the HLJ and BJ populations of *B. b. ussuriensis* and suggested that the two populations were significantly isolated.

*Bubo bubo kiautschensis*, *B. b. tibetanus,* and *B. b. ussuriensis* showed a well-differentiated relationship with a recent divergence. The median-joining network analysis showed a line-like topology, clustered according to geographical origin (Figure 1(B)). This result indicated that individual haplotypes were coordinated with geographic and climate patterns. Furthermore, the Bayes phylogenetic tree constructed for the three subspecies’ haplotype groups yielded two clades that Clade I was well-supported (*PP* = 1.0), but Clade II was poor-supported *(PP* = 0.78) (Figure 1(C)). Above all, these results represented these three subspecies to be independent lineages with a recent divergence.

## Data Availability

The data that support the findings of this study are openly available in the National Center for Biotechnology Information (NCBI) at [https://www.ncbi.nlm.nih.gov/], reference number [MK656273-MK656287].
